# Supporting Autonomous Motivation for Physical Activity With Chatbots During the COVID-19 Pandemic: Factorial Experiment

**DOI:** 10.2196/38500

**Published:** 2023-01-25

**Authors:** Wendy Wlasak, Sander Paul Zwanenburg, Chris Paton

**Affiliations:** 1 Department of Information Science University of Otago Dunedin New Zealand; 2 Centre for Tropical Medicine University of Oxford Oxford United Kingdom

**Keywords:** autonomous motivation, chatbots, self-determination theory, physical activity, factorial experiment, mobile phone, COVID-19

## Abstract

**Background:**

Although physical activity can mitigate disease trajectories and improve and sustain mental health, many people have become less physically active during the COVID-19 pandemic. Personal information technology, such as activity trackers and chatbots, can technically converse with people and possibly enhance their autonomous motivation to engage in physical activity. The literature on behavior change techniques (BCTs) and self-determination theory (SDT) contains promising insights that can be leveraged in the design of these technologies; however, it remains unclear how this can be achieved.

**Objective:**

This study aimed to evaluate the feasibility of a chatbot system that improves the user’s autonomous motivation for walking based on BCTs and SDT. First, we aimed to develop and evaluate various versions of a chatbot system based on promising BCTs. Second, we aimed to evaluate whether the use of the system improves the autonomous motivation for walking and the associated factors of need satisfaction. Third, we explored the support for the theoretical mechanism and effectiveness of various BCT implementations.

**Methods:**

We developed a chatbot system using the mobile apps Telegram (Telegram Messenger Inc) and Google Fit (Google LLC). We implemented 12 versions of this system, which differed in 3 BCTs: goal setting, experimenting, and action planning. We then conducted a feasibility study with 102 participants who used this system over the course of 3 weeks, by conversing with a chatbot and completing questionnaires, capturing their perceived app support, need satisfaction, physical activity levels, and motivation.

**Results:**

The use of the chatbot systems was satisfactory, and on average, its users reported increases in autonomous motivation for walking. The dropout rate was low. Although approximately half of the participants indicated that they would have preferred to interact with a human instead of the chatbot, 46.1% (47/102) of the participants stated that the chatbot helped them become more active, and 42.2% (43/102) of the participants decided to continue using the chatbot for an additional week. Furthermore, the majority thought that a more advanced chatbot could be very helpful. The motivation was associated with the satisfaction of the needs of competence and autonomy, and need satisfaction, in turn, was associated with the perceived system support, providing support for SDT underpinnings. However, no substantial differences were found across different BCT implementations.

**Conclusions:**

The results provide evidence that chatbot systems are a feasible means to increase autonomous motivation for physical activity. We found support for SDT as a basis for the design, laying a foundation for larger studies to confirm the effectiveness of the selected BCTs within chatbot systems, explore a wider range of BCTs, and help the development of guidelines for the design of interactive technology that helps users achieve long-term health benefits.

## Introduction

### Background

Although physical activity is essential for physical and mental well-being, many people struggle to reach recommended levels. For example, in the United States, 76.8% of adults [1] and 85% of adolescents [2] exercise less than the recommended 150 minutes a week. This falls under a broader, long-term trend of falling physical activity levels as advancements in technology continue to transform human occupations [3].

During the COVID-19 pandemic, the need for individuals to undertake regular physical activity has become even more important. By boosting the immune system, physical activity makes people less vulnerable to infections [4,5] and is also associated with a reduced incidence of diabetes, cancer, osteoporosis, cardiovascular disease, and other health conditions that are associated with more severe COVID-19 infection trajectories [6]. Furthermore, physical activity can enhance mental well-being [7,8], which has deteriorated during the pandemic [9,10].

However, during the COVID-19 pandemic, a decrease in physical activity of approximately 50% was found [11,12]. Potentially, this was because of fewer opportunities to be physically active. As the virus spread quickly around the world, governments set up restrictions ranging from social distancing and wearing masks to complete lockdowns, with people being confined to their residences. Many governments closed public facilities such as fitness centers, swimming pools, and parks and restricted access to workplaces, causing a shift toward working from home.

The decline in physical activity and its heightened importance highlight the increased need to support physical activity. With restrictions in place, such support would be most effective if it could be offered independent of recreational facilities and in-person social contact.

### Information Systems as Supporters of Physical Activity

Information systems for physical activity (ISPAs), such as physical activity devices and apps, operate independently of a physical location, require no human contact, and offer several additional benefits. Their ability to reach a wide user base, scalability, and low running costs make ISPAs exceptionally affordable and easily accessible for those seeking physical activity support. These systems are ubiquitous and can automatically capture and use personal and contextual data to provide support at all times and for as long as required.

Chatbots could potentially support physical activity in ways inspired by human-provided support. They range from simple programs that allow the user to choose from predefined answers to intelligent programs that capture sensor data, apply machine learning algorithms, and use natural language processing to imitate human conversation. Although chatbots might not outperform humans in many aspects, such as detecting sentiments or sarcasm, they have been shown to offer options and choices within interactions more consistently than humans [13] and have the advantage of providing anonymity. They can be tested and used in an unbiased and private setting, which is more likely to lead to an adoption among less active individuals who feel socially insecure about their appearance or performance [14].

Chatbots have been used in intervention studies before, targeting a variety of health factors such as physical activity, diet, medication adherence, and mental well-being [15]. A review by Luo et al [15] concluded that chatbots are a promising medium for increasing physical activity. However, the potential long-term impact of using chatbots, or the predictors thereof, has not been well studied. Furthermore, Zhang et al [16] pointed out that there is still a lack of practical recommendations regarding the design of chatbots for behavior change. How can we leverage the literature on behavior change in the design of effective chatbots?

Our overarching objective is to lay a theory-driven foundation for the development of design guidelines for chatbot systems that can help users sustainably improve their physical activity.

### Sustainable Behavior Encouragement

To design ISPAs that effectively support sustained behavior, building on a theoretical foundation is the most viable method [17]. A theory that provides a promising foundation for sustained behavior changes is self-determination theory (SDT) [18]. Similar to many behavior models and theories (eg, Fogg Behaviour Model [19] and Michie’s [20] capability, opportunity, and motivation as three key factors capable of changing behavior [COM-B] model), SDT posits that motivation is important for engaging in a behavior. Furthermore, it posits that long-term behavior is associated with a specific type of motivation, namely autonomous motivation (enjoying or valuing a behavior), and not controlled motivation (feeling pressured) [21,22]. For autonomous motivation to flourish, the basic psychological needs of autonomy (feeling self-directed), competence (feeling capable), and relatedness (feeling connected) must be satisfied. Whether these needs are satisfied or thwarted depends in part on the environment, and the satisfaction of these needs can be deliberately targeted with behavior change techniques (BCTs) in social and technical interventions.

**Figure 1:**
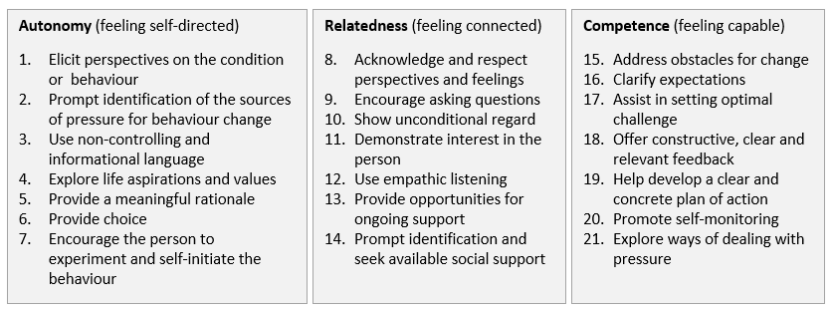
Three categories of motivation and behavior change techniques according to Teixeira et al [28].

Most ISPAs are already consistent with the available BCTs such as providing instructions, providing feedback on performance, and setting goals [23-25]. There are still many more BCTs that could help make ISPAs more effective in changing behavior that are not yet provided within the systems. However, the choice of BCTs must be made carefully to support need satisfaction and, thus, autonomous rather than controlled motivation. Initial evidence [26,27] suggests that the current ISPAs can lead to controlled motivation for physical activity and decreased enjoyment, offering a potential explanation for the high rates of abandonment. Teixeira et al [28] recently proposed a collection of motivation and BCTs (MBCTs), which are a subset of techniques based on SDT. This collection of techniques, as shown in Figure 1, should foster need satisfaction and, hence, autonomous motivation.

Although the use of MBCTs should make environments more need supportive, their effects have yet not been tested in ISPAs. So far, some techniques such as action planning and experimenting are hardly found in ISPAs [29] despite their potential [28,30].

Other techniques such as goal setting have already been implemented in many physical activity apps and devices [23,25]; however, their implementation has not necessarily been based on SDT. For example, MBCT 17 states that support should be provided for setting a goal that is realistic, meaningful, challenging, and achievable [28]. Traditional goal setting usually occurs during the initial setup of the app. The default is often 10,000 steps a day, which is not revised. It can be argued that an alternative goal that is set after a baseline week, focuses on weekly active minutes, and is reviewed weekly fulfills these criteria better. A goal of 10,000 steps a day is unlikely to be met by sedentary people [31], and most ISPAs do not include a function to re-evaluate goals [29]. Although it is sometimes possible to change goals, this is often not done [32].

Furthermore, these techniques can theoretically impact each other. According to SDT, providing individuals with choices to manage their physical activity should satisfy their need for autonomy. However, it is unclear from the literature whether this also means that the users of an ISPA should be given an option not to engage in an MBCT. A good example is action planning. Although action planning should support need satisfaction and motivation, pressuring a user might weaken their feeling of autonomy.

### Study Design

**Figure 2:**
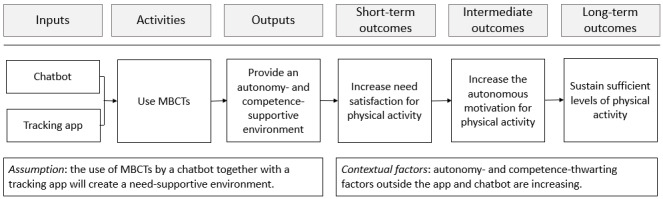
Logic model. MBCT: motivation and behavior change technique.

The overall objective of our study is to evaluate the feasibility of a chatbot system that improves the user’s autonomous motivation for walking based on BCTs and SDT. On the basis of the aforementioned literature, we developed a logic model, which is depicted in Figure 2 and serves as the basis for this study.

The chatbot system itself is composed of a chatbot (implemented on Telegram [Telegram Messenger Inc]) and a tracking app (Google Fit [Google LLC]). This combination enables a basis for implementing various BCTs, including action planning, experimenting, and goal setting. On the basis of the literature, we posit that the implementation of these techniques in the chatbot system can support the user’s sense of competence and autonomy and, thereby, improve need satisfaction and autonomous motivation, a key factor of sustained behavior.

To examine these mechanisms, we tested 3 main hypotheses. First, we hypothesized that the use of a chatbot system can increase autonomous motivation (hypothesis 1 [H1]). Second, we hypothesized that autonomous motivation is positively associated with an ISPA being perceived as supportive of the needs of autonomy and competence (hypothesis 2 [H2]). Third, we hypothesized that the different implementations of MBCTs will have different effects (hypothesis 3 [H3]):

- The use of the chatbot with alternative goal setting based on MBCT 17 (vs traditional goal setting) will be perceived as more supportive (H3a) and lead to higher need satisfaction (H3b) and autonomous motivation (H3c).
- The use of the chatbot with experimenting based on MBCT 7 (vs no experimenting) will be perceived as more supportive (H3d) and lead to higher need satisfaction (H3e) and autonomous motivation (H3f).
- The use of the chatbot with action planning based on MBCT 19 (vs no action planning) will be perceived as more supportive (H3g) and lead to higher need satisfaction (H3h) and autonomous motivation (H3i).
- The use of the chatbot with optional action planning based on MBCTs 6 and 19 (vs mandatory action planning) will be perceived as more supportive (H3j) and lead to higher need satisfaction (H3k) and autonomous motivation (H3l).

## Methods

### Overview

We developed a chatbot system based on the BCTs of action planning, experimenting, and goal setting, deployed this system in a study focused on walking, and tested the above hypotheses within a small sample of 102 users who were not current users of any ISPA.

The chatbot system we developed was based on the mobile apps Telegram and Google Fit. Telegram allowed the greatest freedom for chatbot implementation, and Google Fit was chosen because it is reliable, simple, and provides the necessary physical activity data associated with walking. We focused on walking, as it is the most common physical activity that can be performed by most people without any equipment. Perceived app or chatbot support, need satisfaction, and physical activity were measured weekly, and motivation was measured before and after the intervention.

To explore the effectiveness of the BCTs, the study was conducted in the form of a 2×2×3 factorial design, configuring the chatbot based on the motivational BCTs of goal setting (traditional vs alternative goal setting), experimenting (experimenting tips vs simple walking reminders), and action planning (mandatory vs optional vs no action planning).

### Participants

Eligible participants had to be aged between 18 and 65 years, live in New Zealand, speak English fluently, and struggle to complete 150 minutes of physical activity a week. Furthermore, they had to be unrestricted in walking, regular smartphone users who did not use an ISPA in the last 3 months, and willing to use Google Fit and Telegram for the purposes of the study.

Recruitment ran from March 2 to April 5, 2021, through social media, emails, and flyers at public places such as on campus and in supermarkets. The participants received a supermarket voucher worth NZD $20 (US $12.8) and entered the draw for an additional voucher worth NZD $50 (US $32.0). Overall, the participants were mostly aged between 18 and 44 years (84/102, 82.4%), female (74/102, 72.5%), studying (35/102, 34.3%) or working (54/102, 52.9%), White (55/102, 53.9%), well educated (69/102, 67.6% had a university or polytechnic degree), in a partnership (76/102, 74.5%), and from the Otago region (57/102, 55.9%). Multimedia Appendix 1 provides further details on the demographic data. No imbalances in potential confounders such as demographics were found.

### Ethics Approval

Before entering the study, the participants were asked to provide informed consent. The form we used informed them about the study procedure, collected data, handling of the data, and right to withdraw at any point in time without any disadvantages. Data were stored using pseudonyms to ensure anonymity of the participants. No personal identifiers were retained, and all data were treated confidentially. The participants received a supermarket voucher worth NZD $20 and entered the draw for an additional voucher worth NZD $50. The study was approved by the department of information science and ethics committee of the University of Otago (reference number D20/032).

### Study Procedure

**Figure 3:**
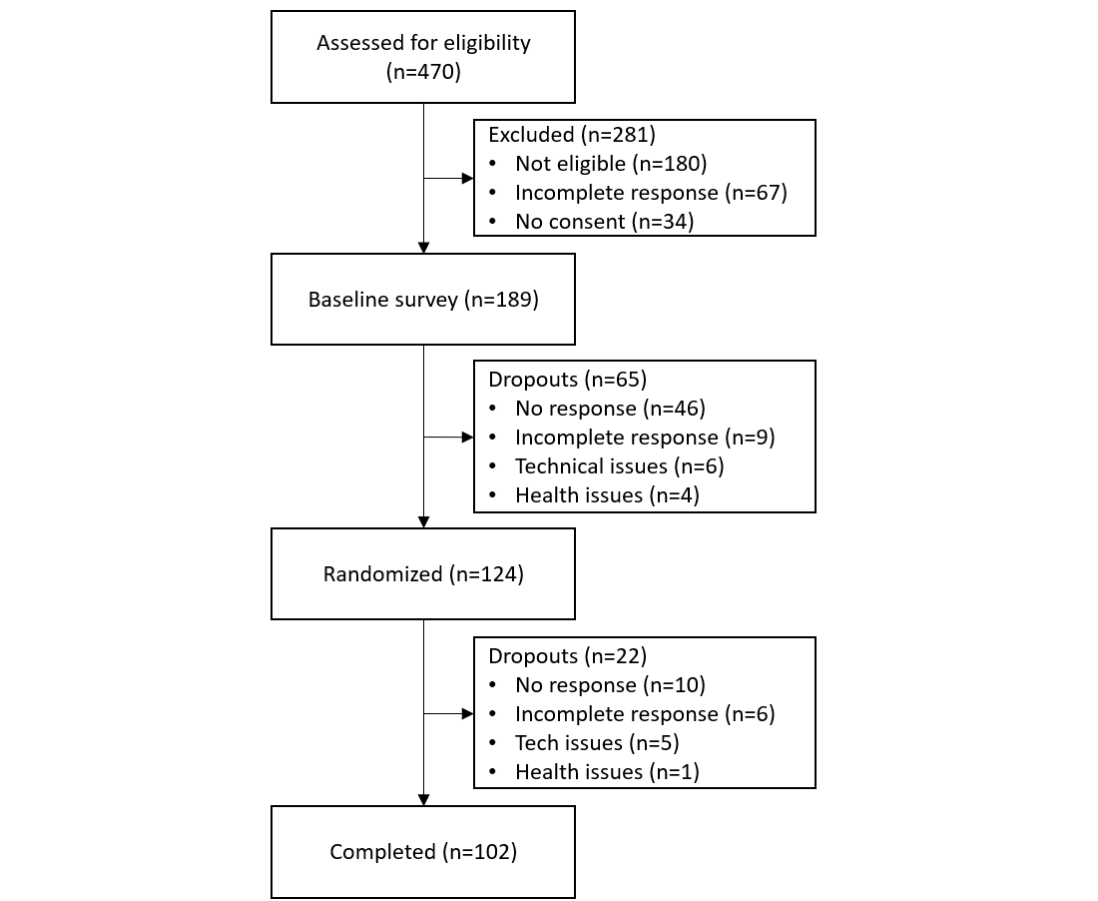
Study recruitment, retention, and analysis.

**Figure 4:**
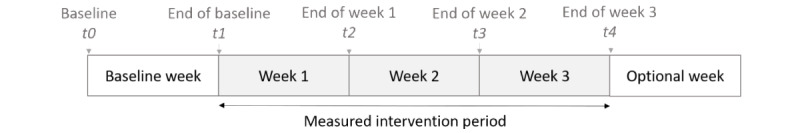
Study timeline.

In total, 470 individuals were assessed for eligibility, of whom 124 (26.4%) completed the baseline survey and were randomized to one of the 12 chatbots. Of these 124 participants, 102 (82.2%) completed the study, and their data were used for analysis, as shown in further detail in Figure 3. The participants had to complete 1 chatbot interaction and 1 survey each week. The study ran for 5 weeks, as illustrated in Figure 4. It started with a baseline week in which Google Fit automatically measured physical activity in the background. This was followed by 3 weeks of intervention in which the participants interacted with the chatbot and Google Fit. After these 3 weeks, the participants had the option to continue using the chatbot for another week.

**Figure 5:**
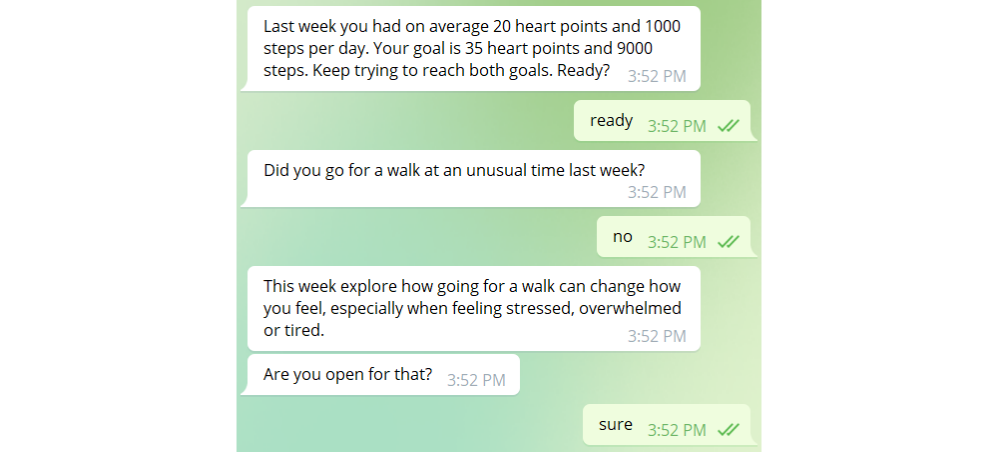
Example of traditional goal setting and experimenting tips (messages from the chatbot on the left and those from the participant on the right).

The chatbot conversations were programmed as follows. On the basis of the group, the chatbot first went through one of the 2 types of *goal setting*. In the alternative goal setting group, the participants were first shown the number of active minutes from the previous week and then prompted to set a challenging but realistic goal for the new week. The traditional goal group also saw the numbers from the previous week but was not prompted to set a weekly goal or review or change their daily goals, as shown in Figure 5.

Then, one-half of the participants received a prompt to experiment with their physical activity behavior, whereas the other half received information that they can increase their physical activity levels by walking more. The *experimenting* group received a different experimenting suggestion each week such as “This week try a new walking route and go somewhere you haven’t been before” or “Try to go for a walk at a *[sic]* for you unusual time for example as first thing in the morning. Maybe that works better for you than you think.”

**Figure 6:**
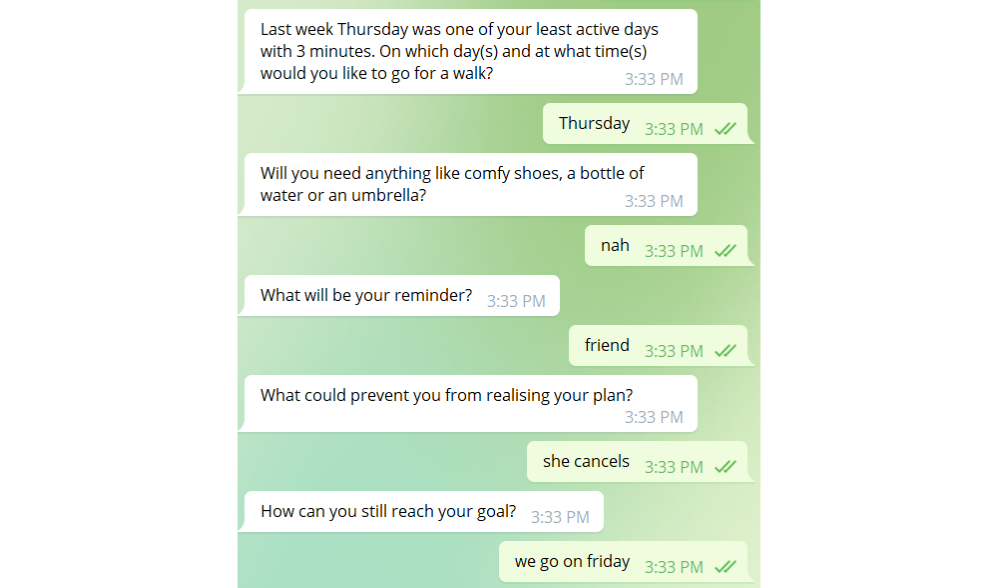
Example of action planning (messages from the chatbot on the left and those from the participant on the right).

Subsequently, one-third of the participants were automatically guided through *action planning*. Another third of the participants were asked whether they wanted to perform action planning, and the remaining third of the participants received no action planning. All the participants were shown which day during the last week was their least active day. The action planning then consisted of 5 parts covering when the participant wants to go for a walk, what they need in preparation, how they remind themselves, possible barriers, and how to overcome them (as shown in Figure 6).

For the chatbot conversations, we used data from Google Fit. Google Fit automatically collects physical activity data through smartphone sensors, measuring steps, move minutes, and heart points (cardio-intensive move minutes), and presents these data in daily and weekly overviews.

### Measures

In this study, we measured the participants’ motivation to walk, need satisfaction for walking, self-efficacy for walking, perceived app support, chatbot impressions, and physical activity.

#### Motivation for Walking

Motivation was measured using an adapted version of the Exercise Regulation Questionnaire [33], which included measures of autonomous motivation, controlled motivation, and individual regulations. The internal consistencies of the autonomous motivation and controlled motivation scales were good, with Cronbach α of ≥.82 across their assessments. The internal consistency of the individual regulations scale was acceptable, with Cronbach α of .71 or higher.

#### Need Satisfaction for Walking

The satisfaction of the needs of autonomy and competence was measured using an adapted version of the Basic Psychological Need Satisfaction and Frustration Scale [34]. The target activity was walking for ≥10 minutes. A total of 16 statements were assessed using a 5-point Likert scale. Internal consistency of the full scale was excellent, with Cronbach α between .90 and .91 for each week. The internal consistency of the subscales was acceptable, with Cronbach α of ≥.72.

#### Self-efficacy for Walking

As walking is a relatively easy activity, there was a concern that the competence measure within need satisfaction might lack variability. Therefore, self-efficacy was included as an additional measure and a potential alternative to the measure of competence. Self-efficacy “...refers to beliefs in one’s capabilities to organize and execute the courses of action required to produce given attainments” [35]. Self-efficacy shares conceptual ideas that are similar to the concept of competence [36]; however, it was expected to show higher variability, as it refers to situational competence. Self-efficacy was measured using the Physical Exercise Subscale of Health-Specific Self-Efficacy Scales from Schwarzer and Renner [37]. It consists of 5 items that relate to self-efficacy by asking “how certain are you that you could overcome the following barriers?” on a 4-point Likert scale. The barriers included being “worried,” “depressed,” “tense,” “tired,” and “busy.” The internal consistency of the scale was good, with Cronbach α of .84 at baseline (*t0*) and 0.86 at the end of the intervention (*t4*).

#### Perceived App Support

The perceived support of the chatbot and Google Fit (combined) was measured using a modified version of the Perceived Environmental Support Scale [38]. The internal consistency of the measure was good, with Cronbach α >.84 for all subscales (structure, autonomy, and involvement) and Cronbach α of ≥.94 for the full scale across all weeks.

We also asked the participants which Google Fit functions they used: whether they entered activities manually, placed a Google Fit widget on their start screen, changed their daily goals, connected Google Fit to another app, or turned off notifications. We also asked them to give a reason for such use, and how supportive they found the Google Fit functions they used and the chatbot functions they were aware of on a 5-point Likert scale ranging from “extremely supportive” to “not supportive at all.”

#### Chatbot Impressions

We asked the participants what they thought about the chatbot using three 5-point Likert items: “It felt like a companion,” “I would have preferred the chat with a human,” “It helped me to get more active.” We further asked about the expected helpfulness (0-5) of a more advanced chatbot, given that the one with which they interacted was basic.

#### Physical Activity

Although the autonomous motivation for physical activity was measured and used as a proxy for sustained physical activity, we also included physical activity measurements to study the immediate impact of the intervention on physical activity in ancillary analysis. In the prescreening survey, physical activity was measured with the short version of the International Physical Activity Questionnaire [39]. During the study, the participants were asked for their move minutes per day and steps and heart points per week, as shown in the Google Fit app.

### Statistical Analysis

Data were analyzed using RStudio (R version 3.6.3; R Foundation for Statistical Computing). For the preliminary analysis, the means and SDs of all scales were calculated for each group. We calculated the median values for steps and move minutes, as they did not follow a normal distribution.

The measure of heart points was excluded from the analysis owing to many participants not receiving any from the app. The measures of steps and move minutes were checked by searching for outliers, based on extremely low or high values and unusual steps/move minute ratios, given that overall, the number of steps and move minutes were strongly correlated. The outliers were followed up with the participants when possible. In total, 4% of the data points were replaced by imputation using linear regression.

Data were first analyzed without separating the different groups to check for overall effects on app support, need satisfaction, self-efficacy, motivation, and physical activity. The baseline values were compared with the values after the intervention. Distributions were checked for normality using Shapiro-Wilk tests and by checking skewness and kurtosis. In the case of a significant deviation from normality, bootstrapped paired 2-tailed *t* test values were calculated. Effect sizes were investigated using Cohen *d*. Furthermore, associations among perceived app support, need satisfaction, self-efficacy, and motivation were investigated through linear regression.

We then investigated each group individually and compared them to the alternative group or groups. In each group, we compared the baseline (*t0*) measures with the measures at the end of week 3 (*t4*) using paired 2-tailed *t* tests. To compare the groups, we tested for differences in demographics and baseline values using chi-square tests for categorical variables and 2-tailed *t* tests for ordinal variables. The 3 action planning groups were compared using 1-way ANOVA.

Given the size of the sample (n=102), the number of relationships, and the exploratory nature of this feasibility study, no path analysis was conducted.

## Results

We have first presented our observations regarding the chatbot system itself and then presented the results regarding the overall intervention. Finally, we have presented support for the underlying theoretical mechanisms and a comparison of the BCT implementations.

### Chatbot Use and Operation

In general, our chatbot system operated satisfactorily. Because it was impossible to automatically extract data from the Google Fit app, we asked respondents to look this up and report it back, which did not affect participation. Relatively few people, that is, of the 189 participants who completed our baseline questionnaire, 11 (5.8%) participants dropped out of our study owing to technical issues outside of our control.

We were able to implement the desired MBCTs by configuring the Telegram chatbot such that it asked people questions on their goal setting or provided suggestions regarding action planning and experimenting.

Overall, the feedback from participants was moderately positive. Most (86/102, 84.3%) participants planned to continue using Google Fit after the study. Approximately, 46.1% (47/102) of the participants stated that the chatbot helped them become more active, whereas 23.5% (24/102) thought that it did not. In some cases, the chatbot felt like a companion (28/102, 27.5% agreed and 48/102, 47.1% disagreed). Almost 50% (50/102) of the participants would have preferred a human over a chatbot to support them, whereas 23.5% (24/102) did not prefer to have a human intervention provider instead. One of the participants explicitly stated that as someone with autism and social anxiety, they preferred the anonymity of the chatbot. The majority (68/102, 66.7%) thought that a more advanced chatbot could be very helpful (3.97 rating out of 5). A total of 42.2% (43/102) of the participants wanted to continue using the chatbot for an additional week.

On the basis of the comments of the participants, the main aspects that caused disappointment with the chatbot were the repetitiveness of dialogues each week and the failure to react to nonstandard (not programmed) responses such as asking questions when no questions were anticipated (which was most of the time). A few participants also stated that they would have liked more interactions with the chatbot.

There was some variability in the use of Google Fit. Most (51/102, 50%) participants did not make use of the optional functions of Google Fit, such as manual tracking, using the start screen widget, connecting other apps, or changing goals, as shown in Table 1. Most participants received 1 or 2 notifications from Google Fit per week, which were mainly goal achievement and goal progress notifications. Goal achievement notifications were perceived as the most supportive.

**Table 1:** Use of the Google Fit functions and their support.

Function	Used or received by (n=102), n (%)	Support
Manual tracking	10 (9.8)	77.8% (7/9) rated it as at least moderately supportive
Google Fit widget	23 (22.5)	85.7% (18/21) rated it as at least moderately supportive
Connection to another app	9 (8.8)	N/A^a^
Change goals	21 (20.6)	N/A
Goal achievement notifications	51 (50)	90.0% (45/50) rated them as at least moderately supportive
Goal progress notifications	39 (38.2)	66.7% (24/36) rated them as at least moderately supportive
Goal change notifications	6 (5.9)	N/A

^a^N/A: not applicable.

In sum, as a system developed for feasibility testing, the chatbot itself performed satisfactorily, indicating the technical feasibility of chatbot systems for physical activity support in general and a willingness to adopt these outside the context of academic studies.

### Impact of the Intervention

**Figure 7:**
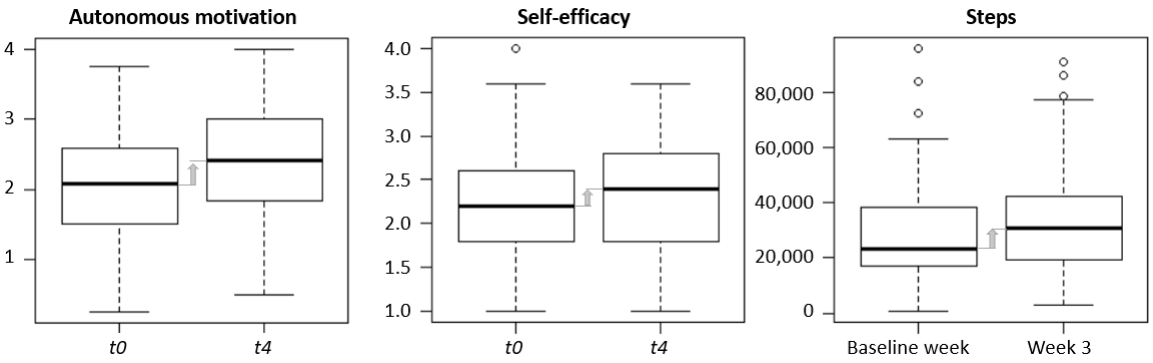
Changes in autonomous motivation, self-efficacy, and physical activity.

We first inspected changes in motivation, self-efficacy, and the number of steps. At baseline (*t0*), motivation was mostly autonomous (mean 2.13, SD 0.78), and controlled motivation (mean 1.41, SD 0.80) and amotivation (*P*<.001; mean 0.86, SD 0.79) were low. After the intervention, autonomous motivation increased (*P*<.001; Cohen *d*=0.54), as shown in Figure 7, whereas controlled motivation and amotivation did not appear to change (Multimedia Appendix 2).

Need satisfaction for walking was high at baseline (*t0*) (mean 2.56, SD 0.66), particularly the satisfaction of the need for competence (mean 2.79, SD 0.85; scale from 0 to 4). There was no clear change in the overall need satisfaction from baseline (*t0*) to the end of the intervention (*t4*). However, the overall satisfaction of the need for autonomy increased (*P*=.04; Cohen *d*=0.19), particularly through a decrease in autonomy frustration (*P*=.04; Cohen *d*=0.20).

As expected, competence in walking lacked variability. Therefore, we studied self-efficacy as a proxy. The results for self-efficacy were mixed, with 45% feeling uncertain about overcoming barriers such as being busy, tired, depressed, tensed, or worried about carrying out walking intentions. Over the course of the study, self-efficacy increased (*P*=.02; Cohen *d*=0.23).

At baseline, the median level of physical activity was 103 minutes per week, as per the International Physical Activity Questionnaire (*t0*). Physical activity measured during the baseline week, as per Google Fit, was approximately 3 times higher, with a median of 355 minutes per week. Physical activity increased during the study, with median weekly steps rising from 23,430 to 30,916 (*P*=.004; Cohen *d*=0.29) and weekly move minutes rising from 272 to 415 minutes (*P*<.001; Cohen *d*=0.53).

Thus, we found support for H1. Autonomous motivation increased after using the chatbot system. Autonomy satisfaction and self-efficacy were also increased. The effect size for the increase in autonomous motivation was medium, whereas the effect sizes for the increases in autonomy satisfaction and self-efficacy were small. No differences were found on the basis of age or sex.

### Test of the Theoretical Mechanisms

**Figure 8:**
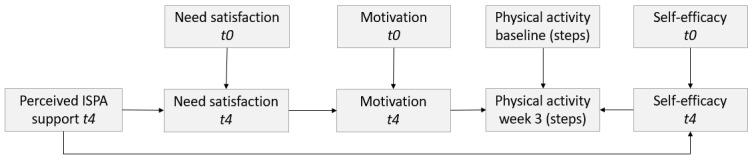
Associations between the key variables.

Figure 8 presents the associations between our key variables of interest, with the following key findings:

- Autonomous motivation at *t4* was associated with the autonomous motivation at *t0* (*P*<.001; *r*=0.35) and need satisfaction at *t4* (*P*<.001; *r*=0.59).
- Need satisfaction at *t4* was associated with the need satisfaction at *t0* (*P*<.001; *r*=0.59) and perceived app or chatbot support at *t4* (*P*=.002; *r*=0.24).
- Self-efficacy at *t4* was associated with the self-efficacy at *t0* (*P*<.001; *r*=0.35) and perceived app or chatbot support at *t4* (*P*<.001; *r*=0.59).
- Self-efficacy was not associated with motivation when competence and autonomy were controlled for.
- Physical activity in week 3 was associated with the physical activity at baseline (*P*<.001; *r*=0.53) and self-efficacy (*P*=.02; *r*=0.20) but not motivation at *t4* (*P*=.59; *r*=0.05).

Hence, we found support for H2 in our study, as autonomous motivation was found to be positively associated with need satisfaction, and need satisfaction was positively associated with perceived app or chatbot support. No association was found between autonomous motivation and immediate physical activity, but a positive association was observed between self-efficacy and immediate physical activity. Furthermore, self-efficacy was positively associated with perceived app or chatbot support.

### Comparison of the Different BCT Implementations

**Figure 9:**
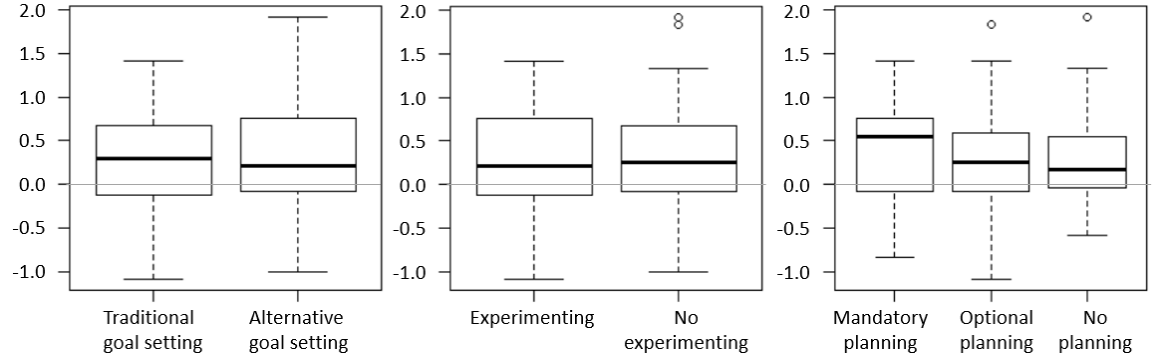
Change in autonomous motivation as compared between groups.

We tested for the differences in implementations, as specified in H3. We found no evidence that alternative goal setting (vs traditional goal setting) was perceived to be more supportive (H3a) or led to greater need satisfaction (H3b) or autonomous motivation (c). In fact, both groups reported positive results: need satisfaction increased marginally in the traditional goal setting group, self-efficacy increased marginally in the alternative goal setting group, and autonomous motivation and physical activity increased in both groups, as shown in Figure 9.

The participants in the experimenting group did not show higher perceived support (H3d) or greater improvements in need satisfaction (H3e) and autonomous motivation (H3f) than the participants who received a reminder to reach their goal by walking more (no experimenting).

The 2 planning groups (optional and mandatory action planning) did not show higher perceived support (H3g) and higher increases in need satisfaction (H3h) and autonomous motivation (H3i) than the no action planning group. Finally, optional actional planning (vs mandatory action planning) did not show higher perceived support (H3j) and higher increases in need satisfaction (H3k) and autonomous motivation (H3l).

To summarize, no evidence was found that any of the alternative implementations were superior to the traditional chatbot implementations in terms of increasing perceived support, need satisfaction, and autonomous motivation. Therefore, H3 could not be confirmed.

## Discussion

### Overview

This study evaluated the feasibility of a chatbot system that improves the users’ autonomous motivation for walking based on BCTs and SDT. We developed and evaluated various versions of the chatbot system based on promising BCTs. We found that the use of the system coincided with increments in the autonomous motivation for walking and the associated factors of need satisfaction. Although we found support for the theoretical mechanism, we did not observe meaningful differences in the effectiveness of various BCT implementations, possibly because of the small sample size.

### Chatbot Implementation

Overall, we received positive feedback from the participants regarding the chatbot system, including their inclination to continue using the chatbot system and Google Fit. Although about half (50/102, 49%) of the participants would have appreciated a human intervention provider instead, also about half (47/102, 46.1%) of the participants thought the chatbot helped them become more active. Approximately, a quarter saw the bot as a companion, and most participants (68/102, 67.6%) saw a lot of potential in a more advanced chatbot. Therefore, the use of chatbots should be investigated further.

Formative feedback from the participant’s experience suggests that future chatbot implementations, in comparison with our own, could provide more interactions to ensure that the bot is able to react to questions and introduce more variability into the interaction to make them more interesting and engaging.

Furthermore, in this study, the need for relatedness—another psychological need specified by SDT—was not specifically targeted, as it has not been shown to be essential for intrinsic motivation in physical activity [21]. However, chatbots could potentially be supportive of relatedness. Involvement support in this study, although not being particularly high, was positive, and the participants often interacted with the chatbot as if it were a human, asking it how it was, proudly sharing their achievements, apologizing for weeks that did not go as hoped, and replying with thank you.

Although future chatbots can also be based on the same platforms of Telegram and Google Fit, this does mean that not every participant will receive the same experience with the system, which can complicate drawing inferences from the data. In our study, most participants (51/102, 50%) did not make use of the additional functions Google Fit and the chatbot provided (the chatbot offered support during the week to, for example, review plans, change goals, and check weather forecasts). Furthermore, we found that the participants received unequal numbers of notifications from Google Fit during the study period. Although some studies have shown that the number and type of notifications can be important [40], the number of notifications did not seem to impact perceived app support in this study.

### Impact of the Intervention

Overall, the intervention was found to have a positive effect on the construct of autonomous motivation (H1). All 3 regulations (intrinsic, integrated, and identified) increased, whereas, as expected, controlled motivation and amotivation did not increase. Given the small sample size and lack of a no-chatbot control group, larger studies or studies with a more specific focus on this particular hypothesis may be able to confirm the statistical significance of these results.

Our findings contrast with some findings in the literature that focus on Fitbit tracking devices. Kerner and Goodyear [27] and Busch et al [41] found a decrease in autonomous motivation and an increase in amotivation. So far, the motivational aspects of chatbots have mostly been tested in smaller qualitative studies [42,43]. Kocielnik et al [42] found that reflective interactions with their chatbot led to an increase in motivation. Future research could investigate whether there is a difference among smartphone apps, chatbots, and tracking devices in terms of their impact on motivation.

Ancillary analysis showed that autonomy frustration decreased and that self-efficacy and physical activity increased significantly. The decrease in autonomy frustration in our study contrasts with the decrease in need satisfaction found in Kerner and Goodyear [27]. However, more recently, Busch et al [41] found a small significant increase in the satisfaction of the need of autonomy in their sample. Physical activity increased significantly, with the average weekly steps rising by 5133 and weekly move minutes rising by 84 minutes, which is similar to the results of other studies that have been successful in increasing physical activity [44]. Previous studies have shown that ISPAs can increase physical activity in the short term [45], particularly when self-monitoring is combined with goal setting [45,46]. Our findings suggest that ISPAs also have the potential to improve the autonomous motivation for physical activity, an important factor for long-term behaviors.

### Test of the Theoretical Mechanisms

In line with SDT, a positive association among perceived app support, need satisfaction, and autonomous motivation was found, with higher perceived app support being associated with higher need satisfaction and higher need satisfaction being associated with higher autonomous motivation. These findings provide further evidence that SDT can function as an effective theoretical basis for designing chatbots that support autonomous motivation (H2).

Although both autonomous motivation and immediate physical activity increased, no association was found between the two variables. There are multiple reasons why this might have been the case; for example, although autonomous motivation increased, not everyone may have had the chance to act on it immediately with the COVID-19 pandemic creating a restricted environment. On the basis of previous literature, it is still expected that autonomous motivation is associated with long-term physical activity, which was not measured in this study.

Physical activity at the end of the intervention was predicted by physical activity at the beginning and self-efficacy at the end of the intervention. The relationship of self-efficacy with physical activity has been demonstrated in previous studies as well [47-49]. Self-efficacy was also found to be a predictor of long-term engagement [50]. The study by Petersen et al [51] provided initial support by showing that the relationship between app use and physical activity was mediated by both autonomous motivation and self-efficacy. Similar to autonomous motivation, self-efficacy at the end of the chatbot intervention was positively associated with perceived app support, underlining the importance of need-supportive ISPAs.

Our findings suggest that chatbots can be effective in increasing autonomous motivation when they are designed to support the basic psychological needs of autonomy and competence.

### Improving the Design of ISPAs

The literature has yet to show how ISPAs can be improved to support long-term physical activity by increasing need satisfaction and autonomous motivation. The use of MBCTs has been suggested as a promising method, but little research has tested these techniques individually. Furthermore, how to implement these techniques most effectively remains an open question.

First, it was hypothesized that the SDT-based alternative goal setting would lead to higher perceived app support (H3a), need satisfaction (H3b), and autonomous motivation (H3c) than the traditional goal setting typically found in ISPAs. However, the 2 forms of goal setting were perceived as similarly supportive. Traditional goal setting did not lead to more need frustration, but autonomy frustration decreased significantly. This might be owing to participants already being moderately active at the beginning of the intervention, and the majority (31/50, 62%) choosing low daily goals (20 heart points and 5000 steps). Motivation did not differ significantly between the groups, with both showing significant increases in autonomous motivation. This suggests that both goal setting versions were similarly effective.

We also hypothesized, based on MBCT 7, that the use of experimenting in ISPAs would lead to higher perceived app support (H3d), need satisfaction (H3e), and autonomous motivation (H3f). Providing experimenting tips in the form of ideas on how to integrate more activity into every life and trying new things did not lead to higher need satisfaction and autonomous motivation. So far, experimenting as a technique has not received much attention; hence, insight into how to provide it is limited. Alternative methods such as learning goals [52] might lead to better results. An example of such a goal is to find 5 ways to increase the daily step count, which ultimately requires experimenting. Learning goals are suspected to be particularly helpful for beginners (in our case, inactive people), as they focus on the ways and means to achieve a goal [53].

On the basis of MBCT 19, we hypothesized that action planning (vs no action planning) in ISPAs would lead to higher perceived app support (H3g), need satisfaction (H3h), and autonomous motivation (H3i). However, action planning did not lead to significantly higher perceived app support, need satisfaction, or motivation. This is surprising, as it has shown promising results in other studies [30]. It is possible that action planning, as implemented in this study, did not achieve its full potential. Some participants may have rushed through the conversations or may not have taken the questions seriously. For example, when asked for a specific time and day to go for a walk, some answered “anytime” or “not sure.” A few participants stated that they had no barriers or that they did not know how to overcome them. These participants may have needed more support, which would have required a more advanced chatbot implementation that reacted to more keywords. Another aspect that potentially frustrated the participants was that the chatbot did not provide reminders, which was something that multiple participants asked for. In addition, repetition of message content was mentioned as a downside of the interactions, which has been reported as an issue in other studies as well [15]. Future research could investigate whether action planning performed with an ISPA can be as effective as action planning performed together with a human intervention provider.

Finally, it was hypothesized that providing optional action planning leads to higher perceived app support (H3j), need satisfaction (H3k), and autonomous motivation (H3l) because it preserves the autonomy of the ISPA user. It was expected that mandatory action planning might be detrimental to the overall autonomy satisfaction, as participants are forced to go through it. For example, Roy and Zaman [54] pointed out in their heuristic that obligatory use should be avoided. Optional action planning did not lead to higher perceived app support, need satisfaction, or motivation. The opposite was the case, with the mandatory planners showing larger increases in need satisfaction, self-efficacy, autonomous motivation, and physical activity. However, between-group differences were not significant. This suggests that making the MBCT mandatory did not harm autonomy satisfaction. However, further studies to confirm this finding should be considered.

Therefore, we found no support for H3. If there were any effects, they were too small to be detected in this small-scale study. Statistically significant effects might be achievable by combining techniques; for example, perceived app support was statistically significantly less when no action planning was combined with no experimenting. However, simply adding up techniques might not always lead to a positive effect; for instance, experimenting without action planning had higher perceived app support scores than experimenting with action planning. This indicates that combinations of techniques should be carefully investigated.

Hence, for the 3 MBCTs that we tested, we found little guidance on their effective implementation within chatbots. Alternative implementations of the same MBCTs may be more effective. Furthermore, there are more MBCTs and combinations of MBCTs that can be studied. These studies will help translate the established theory into design principles for chatbots that can aid in sustained behavior change.

### Limitations

This study was limited by time constraints, the lack of inclusion of a general control group, and the sample size. To directly measure the long-term effects of an intervention on physical activity, a study period of at least 6 months would have been needed. As this was not deemed feasible for this study, we opted for a well-established predictor of sustained physical activity: autonomous motivation. We expect that future studies would again confirm that increases in autonomous motivation would predict increases in physical activity.

This study did not include a general control group that did not use the chatbot. Because our focus was to study whether and how an SDT-based chatbot could improve autonomous motivation for physical activity, we included control groups using versions of the chatbot. We found no substantial basis for expecting that there would be any unobserved impact on autonomous motivation over the course of the 4-week period; future studies that adopt a general control group would be able to test this.

The sample size of 102 participants limited our ability to detect very small effect sizes but can be considered reasonable to gain insights into this feasibility study. Schoeppe et al [55] investigated 27 studies that tested the efficacy of app interventions for dieting, physical activity, or sedentary behavior, which were mostly randomized controlled trials. Of these 27 studies, 20 (74%) had <100 participants. The small sample size also means that the results might not be generalizable to the overall population.

### Conclusions

This feasibility study investigated the use of chatbots as supporters of sustained physical activity by testing different chatbot variations. It presents an example of how chatbots and BCTs could be studied in greater depth. It provides promising initial evidence that a chatbot combined with a tracking app such as Google Fit can increase autonomous motivation, which has been shown to be important for long-term behavior maintenance, by supporting the basic needs of autonomy and competence. Although it remains unclear whether and how particular implementations of BCTs can be best leveraged for improving autonomous motivation, our study identifies various suggestions for an improved design of chatbot systems in terms of the quantity of interactions, responsiveness, and variability within the chatbots. Further research is needed to test more techniques and alternative implementations to strengthen the basis for the design of chatbots that support sustained behavior changes.

## Appendix

Multimedia Appendix 1 Demographics of the total sample and the subgroups, with *P* values indicating whether the groups were significantly different in any of the characteristics.

Multimedia Appendix 2 Overview of the key variables measured at baseline and at the end of the intervention for the total sample and the subgroups, with *P* values comparing the measures before and after the intervention within the subgroups. Cronbach α for the t4 questionnaires.

## Abbreviations

BCT: behavior change technique
COM-B: capability, opportunity, and motivation as three key factors capable of changing behavior
H1: hypothesis 1
H2: hypothesis 2
H3: hypothesis 3
ISPA: information system for physical activity
MBCT: motivation and behavior change technique
SDT: self-determination theory
